# Massive Solubility Changes in Neuronal Proteins upon Simulated Traumatic Brain Injury Reveal the Role of Shockwaves in Irreversible Damage

**DOI:** 10.3390/molecules28196768

**Published:** 2023-09-22

**Authors:** Amir Ata Saei, Hassan Gharibi, Hezheng Lyu, Brady Nilsson, Maryam Jafari, Hans Von Holst, Roman A. Zubarev

**Affiliations:** 1 Division of Physiological Chemistry I, Department of Medical Biochemistry and Biophysics, Karolinska Institutet, 171 65 Stockholm, Sweden; amirata.saei.dibavar@ki.se (A.A.S.); hassan.gharibi@ki.se (H.G.); hezheng.lyu@ki.se (H.L.); brady.ilsson@gmail.com (B.N.); maryam.jafari@ki.se (M.J.); 2 Department of Cell Biology, Harvard Medical School, Boston, MA 02115, USA; 3 Division of Clinical Neuroscience, Section of Neurosurgery, Karolinska Institutet, 171 65 Stockholm, Sweden; 4 Department of Pharmacological & Technological Chemistry, Sechenov First Moscow State Medical University, 119146 Moscow, Russia; 5 The National Medical Research Center for Endocrinology, 115478 Moscow, Russia

**Keywords:** acceleration, biomarker, biomaterial, neuron, PISA, shock meter, proteomics, thermal profiling

## Abstract

We investigated the immediate molecular consequences of traumatic brain injuries (TBIs) using a novel proteomics approach. We simulated TBIs using an innovative laboratory apparatus that employed a 5.1 kg dummy head that held neuronal cells and generated a ≤4000 g-force acceleration upon impact. A Proteome Integral Solubility Alteration (PISA) assay was then employed to monitor protein solubility changes in a system-wide manner. Dynamic impacts led to both a reduction in neuron viability and massive solubility changes in the proteome. The affected proteins mapped not only to the expected pathways, such as those of cell adhesion, collagen, and laminin structures, as well as the response to stress, but also to other dense protein networks, such as immune response, complement, and coagulation cascades. The cellular effects were found to be mainly due to the shockwave rather than the g-force acceleration. Soft materials could reduce the impact’s severity only until they were fully compressed. This study shows a way of developing a proteome-based meter for measuring irreversible shockwave-induced cell damage and provides a resource for identifying protein biomarkers of TBIs and potential drug targets for the development of products aimed at primary prevention and intervention.

## 1. Introduction

Traumatic brain injury (TBI) is a serious global health problem [1] that affects around 70 million individuals every year [2]. It is sometimes called the “silent epidemic” because many cases are not registered and, thus, are not reflected in official statistics [3,4]. Not only TBI victims but also their relatives suffer significantly, and the economic burden on society is, therefore, very large [5,6]. Over the last decades, several studies have found and confirmed that one of the more serious consequences of TBI is dementia, even in younger individuals [7]. Recently, it was reported that 6.3% of TBI cases were associated with dementia within 15 years after an impact to the head [8]. With the increasing interest in risky leisure activities worldwide, the number of dementia cases following TBIs is expected to spike dramatically during the next decades. Although society in general has understood the advantages of primary prevention, such as using helmets, more efforts are required to reduce the severe consequences of TBI. Furthermore, conventional helmets do not always protect from the severe consequences of head impacts, as the well-known accident with Michael Schumacher has proven.

Today, the treatment of moderate or severe TBIs is still challenging. The most significant predictor of patients’ outcomes is the development of cytotoxic brain tissue edema, which is defined as a substantial increase in intracellular water content that leads to an expansion of brain volume [9]. When this is severe enough, it causes increased intracranial pressure, which is usually refractory to existing osmotic treatments. The ultimate choice of therapy is decompressive craniotomy, which diminishes intracranial hypertension [10]. While this surgery significantly reduces the mortality rate, it creates further complications [11,12].

A number of the molecular mechanisms underlying TBI are already known, but the molecular etiology of TBI and its effects at the cellular level are not completely understood. TBI involves the two stages of primary and secondary injury. While the primary injury occurs during the direct insult to the brain, the secondary injury happens through subsequent biochemical changes, such as ischemia, inflammation, and cytotoxic processes [13]. The main secondary TBI-induced injuries include Wallerian degeneration of axons, which is defined as the disorganization of the axonal cytoskeletal network [14], mitochondrial dysfunction [13,15,16], excitotoxicity through excessive release of excitatory amino acids [17,18], neuroinflammation through infiltration of circulating lymphocytes, neutrophils, and monocytes into the injured brain parenchyma [19], oxidative stress [15,20] and consequent lipid peroxidation [21], impairment of autophagy and lysosomal pathways [22,23,24,25], and the apoptotic cell death of neurons, oligodendrocytes, and glia [26,27,28].

Extensive research has been conducted to identify druggable targets associated with these processes in order to reduce and possibly prevent the adverse effects of TBI. However, most research has been focused on second-generation primary intracellular phenomena, such as the excessive release of excitatory glutamate and aspartate from presynaptic nerve terminals [17], while first-generation primary processes that occur in brain cells immediately after impact have received significantly less attention. Potential therapeutics have been centered on stabilizing the site of injury and preventing secondary damage. For example, glutamate receptor antagonists (for the protection of neurons against excitotoxicity), antioxidants, anti-inflammatory and anti-apoptotic agents, neurotrophic factors, and stem cell therapies have been considered [13]. However, our limited understanding of the pathophysiology of TBI and the focus on secondary effects have hampered the development of effective TBI treatments. During the last three decades, more than 30 diagnostics or therapeutic pharmaceutical agents for TBI have failed in clinical trials [13,29]. The primary injuries are believed to be hardly reversible [13]; however, the history of biology and medicine contains examples of the successful reversal of phenomena that were deemed irreversible by previous research; e.g., cell differentiation [30].

Therefore, in the current study, we focused on the first-generation primary molecular phenomena in TBI. Our working hypothesis was that external impact causes protein unfolding, which triggers all subsequent phenomena in TBI. It is well known that high static pressure can unfold proteins [31]. Previously, by using computer simulations and mechanistically simulated impact, we showed that a dynamic pressure pulse can affect the aggregation state of laminin LN521, which was chosen as a representative protein due to its high abundance in cells [32]. To study such effects at the level of the whole proteome, here, we employed a previously designed apparatus [32] and simulated a dynamic impact on human-brain-derived cells. The apparatus included a cell culture plate with human neuroblastoma SH-SY5Y cells and allowed for a 5.1 kg dummy head to fall from different heights onto a static bottom plate (floor), thus inducing (depending on the height of the impact) up to 4000 g acceleration, which was measured with a computer-controlled sensor. To measure the effect of the impact on proteins’ aggregated state, we employed the recently introduced Proteome Integral Solubility Alteration (PISA) [33] assay, which is the high-throughput version of Thermal Proteome Profiling (TPP) [34,35,36]. PISA can monitor the solubility changes that occur in the whole proteome due to a variety of cell-stimulating influences [37,38,39,40,41,42].

The dynamic impact of the dummy head holding the cell culture plate against the static bottom plate generated a pressure wave that was characterized by the maximum acceleration (which can be measured in g) and the speed of propagation. The propagation speed is essential for the damage that a wave creates. The initial wave caused by the impact, which is known as a shockwave [43], is supersonic in a given material and deposits energy in the material through which it passes, causing damage at both the molecular and supramolecular levels. A shockwave is responsible for, e.g., the well-known phenomenon of the erosion of hard material by water droplets that fall on it with a speed as low as <3 m/s [44]. After losing substantial energy, the shockwave slows down to the speed of sound, and the resulting acoustic wave propagates causing less damage at the molecular level, even though it may exert significant transient pressure. As we could measure only the acceleration (g) experienced by the cell culture but not the speed of the pressure wave passing through it, we had to untangle the effect of the shockwave on cells from the effect of acceleration.

## 2. Results

### 2.1. The Dynamic Impact Experiments

**Figure 1:**
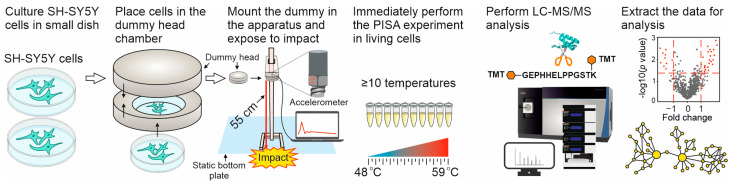
Workflow of the dynamic impact simulation. See the text and methods for explanations. The dummy head holding neuronal cells fell on the static bottom plate (floor) with or without an energy-absorbing material.

The workflow of the dynamic impact experiment is shown in Figure 1. The SH-SY5Y neuroblastoma cells were cultured in Nunclon dishes (diameter 35 mm; height 10 mm) to a density of 250,000 cells per dish, and the dishes were placed in a 5.1 kg dummy head that was dropped from different heights (55–110 cm) onto the static bottom plate (hard surface). In some experiments, the static bottom plate was covered with a mat composed of energy-absorbing material (a rubber made of isobutylene and isoprene designed in the form of pyramids with a length of 6 mm connected to a base membrane with a thickness of 2 mm). An ICP shock accelerometer (PCB Piezotronics, SHEAR ICP shock accelerometer model 350D02 with a sensitivity of ±1 g) was attached to the top of the dummy head. The PicoScope 6 software v6.12.5 was used to record the g-force time diagram during the impact. Both the impact-treated cells and the control cells that were not exposed to the impact were then used for viability measurements and PISA analysis immediately after the impact [33]. Alternatively, cells were kept in culture for 24–48 h for subsequent viability measurements. PISA analysis of the cells exposed to impact vs. the control cells yielded information on the changed solubility of the proteins. As all experiments were performed in several replicates, the statistical significance of each protein’s solubility changes was calculated by using a two-tailed unpaired Student *t*-test. The most significantly changed proteins were mapped on known signaling and metabolic pathways.

### 2.2. Acceleration during Impact

**Figure 2:**
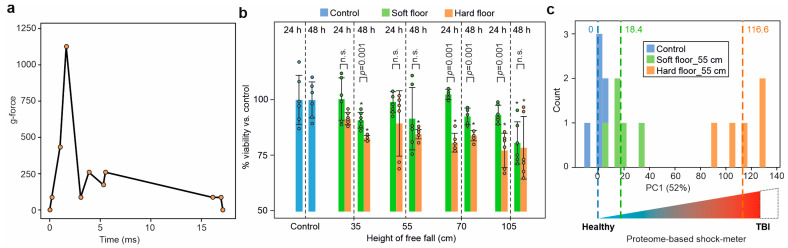
The effects of the dynamic impact simulation on the viability and proteome of SH-SY5Y cell with and without the energy-absorbing mat. (**a**), A representative g-force time diagram for the 55 cm dynamic impact. (**b**) Height-dependent loss of cell viability in response to dynamic impact at 24 and 48 h. Protective effect of the energy-absorbing mat against the loss of cell viability in response to the dynamic impact at increasing heights (six biological replicates; results are shown as mean ± SD; asterisks denote *p*-value < 0.05 vs. the control for the same timepoint; two-sided Student *t*-test). (**c**) PCA component PC1 of the PISA proteome signatures for the controls and cells exposed to a dynamic impact with and without the energy-absorbing mat (five biological replicates). The count refers to the number of samples in a given position along PC1. A conceptual schematic of the proteome-based shock meter is shown.

The g-force during impact was determined in a series composed of ≥4 independent measurements. On average, the impact from the 55 cm height on the hard static bottom plate produced an acceleration of 1556 ± 456 g, while the impact from the 110 cm height produced an acceleration of 3959 ± 978 g. For the static bottom plate with the energy-absorbing material, the corresponding values were 900 ± 289 g and 1625 ± 510 g for 55 cm and 110 cm heights, respectively. A representative g-force time diagram for the hard 55 cm impact is shown in Figure 2a.

### 2.3. Dynamic Impact Reduced Cell Viability

After the dynamic impact, the cells were kept in culture for 24 h and 48 h, after which measurements showed reduced cell viability in a height-dependent manner compared to the control at both timepoints. Notably, 48 h after the hard impact, the cell viability diminished compared to that at 24 h, indicating the persistence of cell death processes initiated by the impact (Figure 2b). The energy-absorbing mat significantly reduced the effect of the dynamic impact on cell viability loss up to a height of 70 cm, but at 105 cm, the viability after 48 h was statistically indistinguishable for the hard and soft impacts (Figure 2b). The Trypan Blue dye exclusion assay showed no significant changes in the percentage of dye-permeable cells upon impact, indicating that the cell membrane remained largely intact, and thus, the cell viability loss was due to internal cell damage.

### 2.4. Dynamic Impact Induced Massive Solubility Changes in the Cellular Proteome

To investigate the mechanism of internal cell damage, the cells exposed to the dynamic impact on the hard surface and the soft surface with energy-absorbing material from a height of 55 cm, as well as the control cells, were immediately processed after the impact for a PISA assay in five biological replicates. In total, 6708 proteins were quantified by using LC-MS/MS. After removing the contaminants and missing values, the final data contained 5612 proteins (Table S1).

At first glance, 25% of the proteome (1382 proteins) showed a significant change (at a *p*-value cutoff of 0.05 and −0.5 > log2 fold change > 0.5) in solubility after the hard impact, while only 1.5% of the proteome (86 proteins) showed a similar change after impact on the plate covered with energy-absorbing material. However, counting significant proteins is a less robust way of quantifying the proteome changes than using the coordinates in principal component analysis (PCA). Previously, we used such an approach in order to quantify the transition of the cellular proteome from stemness to differentiation [38].

When the impact PISA data were subjected to PCA, the first principal component (PC1) accounted for the main part (52%) of data variation and separated the data points by the harshness of the impact, as expected (Figure 2c). If the average position on PC1 of the control cells was taken as zero, the average coordinate of the cells after the soft-floor impact would be 18.4 ± 13, and that of the hard-floor impact would be 117 ± 15. Therefore, PC1 of the PCA plot of the PISA data can be used as a “shock meter” for quantitatively assessing the effect of a given dynamic impact on the cells. However, later analysis showed the necessity of differentiating between the protein markers of reversible and irreversible cell damage.

### 2.5. Individual Protein Solubility Changes after Dynamic Impact

**Figure 3:**
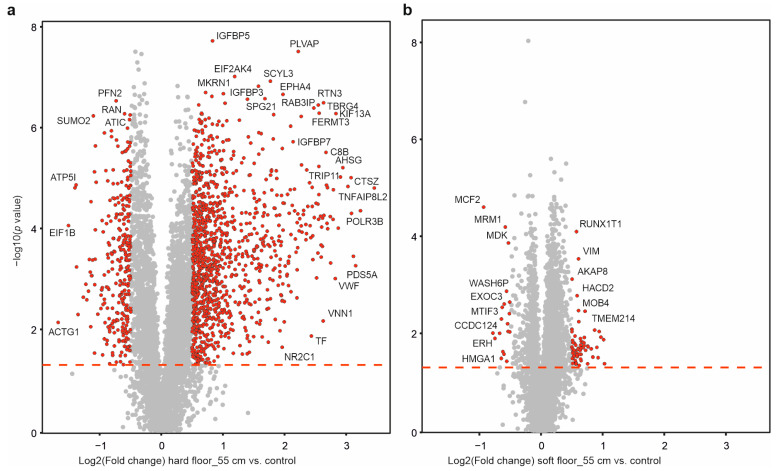
The extent of solubility changes across the proteome upon hard and soft impacts. The plot of the protein fold change (vs. control) against significance in the PISA assay highlights the proteins with solubility/stability changes in response to dynamic impacts on the hard (**a**) and soft surfaces (**b**) with a drop from 55 cm. The most important outliers (absolute log2 fold change vs. control > 0.5 and *p*-value < 0.05) are highlighted (five independent biological replicates). The chosen cutoff is arbitrary.

In the volcano plots in Figure 3a,b, the proteins that changed their solubility significantly upon dynamic impact are shown in red. There were massive changes in protein solubility upon the hard impact, and they were alleviated to a large extent by the energy-absorbing material.

**Figure 4:**
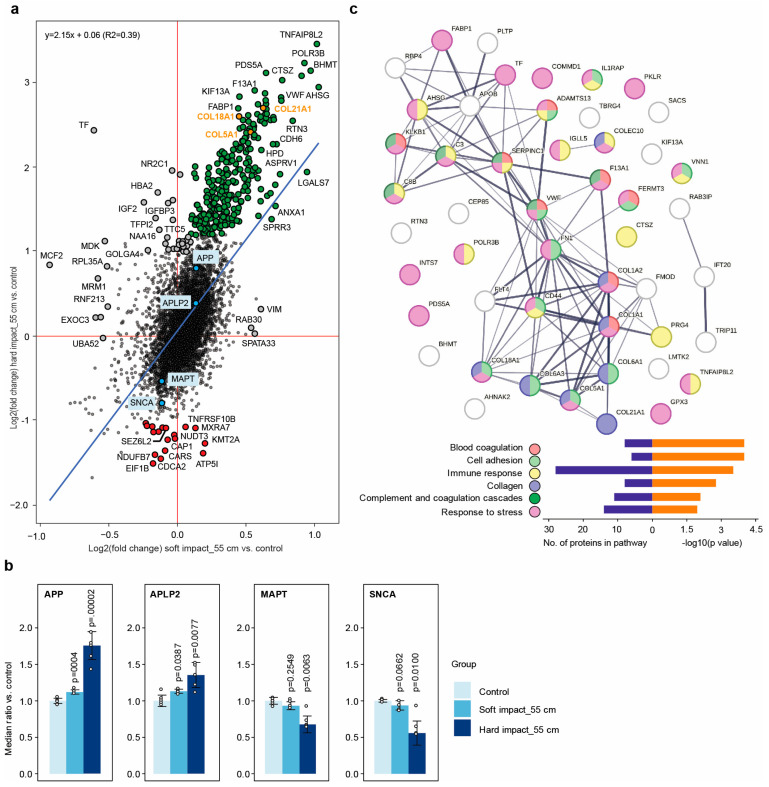
The massive proteome solubility changes affected essential cellular pathways. (**a**) Comparison of the proteome response under the two impact conditions. The proteins of special interest (see text) are shown in green (solubility increased), orange (collagens, solubility increased), red (solubility decreased), and sky blue (proteins known to be involved in the pathology of neurodegenerative diseases). (**b**) Changes in the solubility of proteins involved in the pathology of Alzheimer’s and Parkinson’s disease upon dynamic impact vs. control. (**c**) Selected gene ontology pathways enriched for the top 50 proteins (see text).

The comparison between the hard and soft impacts in Figure 4a shows that mostly the same proteins were affected in both cases, but the change in solubility was much larger upon the hard impact than that upon the soft impact. Inspecting the proteins in Figure 4a can be helpful in the identification of potential diagnostic and/or therapeutic targets. Of particular interest is the group of proteins that changed their solubility in the same direction upon both hard and soft impacts, but with a greater solubility change upon the hard impact. Such proteins are highlighted in green and orange in Figure 4a (the annotations can be found in Table S1). The top protein in that group was tumor necrosis factor alpha-induced protein 8-like protein 2 (TNFAIP8L2), which is involved in maintaining immune homeostasis by preventing the hyperresponsiveness of the immune system [45,46]. Among the top proteins of interest, collagens COL5A1, COL18A1, and COL21A1 had the laminin G domain (shown as orange dots), which is similar to laminin LN521, which was previously found to unfold upon dynamic impact according to electrophoresis and electron microscopy [32].

There was also a distinct group of proteins with reduced solubility (likely to become unfolded) upon dynamic impact (red dots in Figure 4a; the annotations can be found in Table S1). Four such proteins (i.e., ATP synthase subunit E (ATP5I), cytochrome b-c1 complex subunit 6 (UQCRH), NADH dehydrogenase [ubiquinone] 1 beta subcomplex subunit 7 (NDUFB7), and succinate dehydrogenase [ubiquinone] cytochrome b small subunit (SDHD)) mapped to the mitochondrial respirasome or electron transport change in the inner mitochondrial membrane (*p* = 0.01). Interestingly, Seizure 6-like protein 2 (SEZ6L2) was also unfolded upon impact. SEZ6L2 contributes to specialized endoplasmic reticulum functions in neurons and is involved in the regulation of neuritogenesis or neuron outgrowth [47,48]. Autoantibodies against SEZ6L2 have been found in several patients with various forms of ataxia with atypical parkinsonism, which usually occurs due to brain injury in regions controlling muscle coordination, such as the cerebellum [49,50].

### 2.6. Dynamic Impact Modulated the Solubility of Proteins Involved in Neurodegenerative Disorders

Neurodegenerative disorders, such as Alzheimer’s and Parkinson’s diseases, are associated with the unfolding and aggregation of the amyloid β and alpha-synuclein (SNCA) proteins, respectively. In our dynamic impact experiment, we observed solubility changes for amyloid precursor protein (APP), amyloid beta precursor-like protein 2 (APLP2), microtubule-associated protein tau (MAPT), and SNCA (Figure 4b). The solubility (or stability) of APP and APLP2 increased, while that of MAPT and SNCA decreased upon the hard and soft dynamic impacts. Interestingly, the dynamic impact on the soft floor also changed the solubility of the above proteins in the same direction as the hard impact, but to a much lesser extent.

An interesting question is that of whether these solubility changes induce long-term consequences in neurons; if yes, this observation could link TBI to neurodegenerative disorders. Since neurons do not undergo cell division, they might accumulate such changes over a lifetime. Given that cellular repair systems, such as autophagy, are compromised in these diseases [51,52], these solubility changes may contribute to or even trigger neuronal degeneration.

### 2.7. Dynamic Impact Affected Essential Cellular Processes

When the top 50 proteins with the most significant changes upon hard impact were subjected to a network analysis in StringDB, they mapped not only to the expected pathways, such as cell adhesion, collagen trimer, and response to stress, but also to other dense protein networks, such as immune response, complement, and coagulation cascades (Figure 4c).

Coagulation factor XIII A chain (F13A1), von Willebrand factor (VWF), complement C3 beta chain (C3), antithrombin-III (SERPINC1), plasma kallikrein (KLKB1), and complement component C8 beta chain (C8B), which are involved in the complement and coagulation cascades, were among the proteins of interest in Figure 4a. The presence of immune response, complement, and coagulation cascades among the affected pathways is noteworthy and, perhaps, indicates that such proteins are evolutionarily designed to immediately respond to trauma, as there is not enough time for protein synthesis in response to imminent stress. The complement system is known to exert robust, immediate, and nonspecific immune responses [53]. In single-cell organisms, C3 provides constant surveillance of invading microbes by inducing protective inflammasome-type responses [54]. It has, therefore, been hypothesized that C3 is part of an ancient evolutionary system and is stored in vesicles, serving as an intracellular defense mechanism that is capable of quick release in defense against pathogenic invaders [55]. C3 and other proteins involved in coagulation processes that were picked up in our analysis are apparently involved in sensing mechanical trauma. Such proteins may, therefore, be investigated as potential biomarkers of severe TBI. It should also be noted that some collagens, such as COL1A1 and COL1A2, are involved in blood coagulation [56] and its activation [57,58], and they can, therefore, trigger secondary damaging phenomena in TBI.

We also investigated the cellular components that were enriched among the proteins of interest. Among the most relevant components to which the top 50 proteins were mapped were the endoplasmic reticulum (17 proteins), extracellular matrix (16 proteins), secretory vesicle (13 proteins), Golgi apparatus (12 proteins), and cytoplasm (42 proteins) (all *p*-values < 0.02). These results may contain clues about the mechanism of primary cellular damage upon dynamic impact.

### 2.8. The Cellular Effects Were Mainly Due to the Impact’s Shockwaves

**Figure 5:**
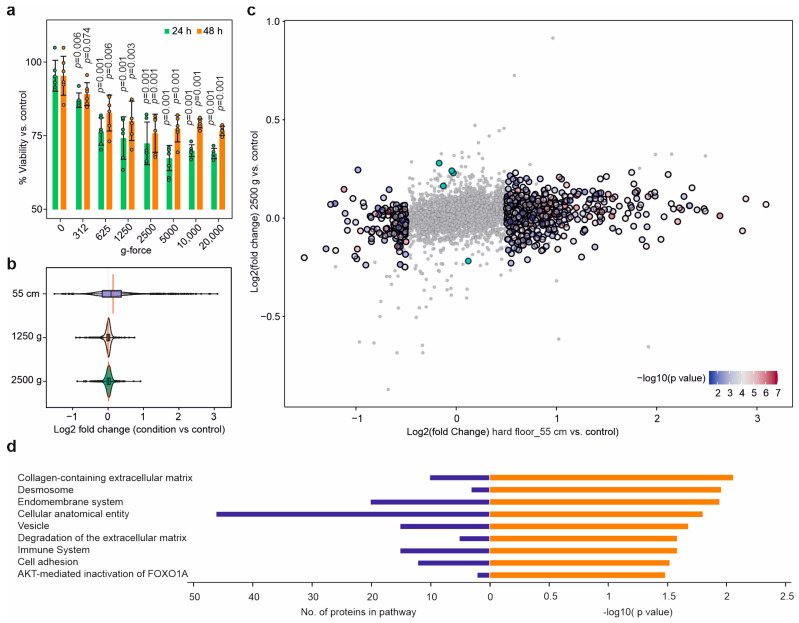
Exposing cells to centrifugal g-force acceleration reduced cell viability in a partially reversible manner and induced only minimal changes in the solubility of the proteome. (**a**) Reduction in cell viability upon centrifugation of SH-SY5Y cells in PBS for 10 min at different g-forces (six biological replicates; *p*-values between the given g-force vs. control; two-sided Student *t*-test). (**b**) The extent of overall proteome solubility change in response to the hard impact (55 cm) vs. centrifugation for 10 min at 1250 and 2500× *g*. (**c**) Solubility changes in response to the hard impact (55 cm) vs. centrifugation for 10 min at 2500× *g*. The chosen cutoff is arbitrary. (**d**) The enriched pathways and processes for the top 50 proteins only changed significantly in response to the hard dynamic impact (and not centrifugation at 2500× *g*).

To investigate whether the cellular effects upon dynamic impact were mostly due to g-force acceleration or shockwaves, SH-SY5Y cells suspended in PBS were exposed for 10 min to centrifugal force equivalent to that experienced upon dynamic impact, after which the cells were plated back and kept in culture, and their cell viability was measured after 24 h and 48 h. At centrifugal g-forces (around 1250) similar to those experienced in the hard dynamic impact at 55 cm and soft impact at 110 cm, the reduction in cell viability after 24 h was also similar (Figure 5a). However, the cell viability did not significantly decrease even at a centrifugal acceleration of 20,000× *g*, and, remarkably, the viability at 48 h increased (vs. controls) compared to that at 24 h for all centrifugal forces, indicating that at least part of the cellular damage was reversible. Note also that the dynamic impact occurred on the millisecond time scale, while the centrifugation was performed over 10 min.

To separate the effects of acceleration vs. shockwaves on the cell proteome, we subjected the centrifuged cells to PISA analysis, merged the datasets for 1250 and 2500× *g*, and compared them with the PISA data from the dynamic impact at 55 cm on the hard floor. The changes in proteome solubility upon dynamic impact were drastically larger than those in cells exposed only to comparable accelerations (Figure 5b,c). Figure 5d shows pathways that were specifically enriched for the set of the top 50 proteins that changed in response to the dynamic impact and not centrifugation. These included expected pathways, such as those of cell adhesion and the immune system, as well as cellular anatomical features.

On the other hand, only five proteins showed a significant change in solubility upon centrifugation at 2500× *g*, with no changes upon dynamic impact from 55 cm on the hard floor. These proteins did not map to any specific pathways.

## 3. Discussion

Here, we demonstrated a new methodology for the simulation of TBI using cultured neuronal cells. Exposing cells to centrifugal g-forces had an effect on cell survival after 24 h that was similar to that of a dynamic impact with an equivalent g-force, even though a much larger magnitude of the effect was expected given the six-order-of-magnitude difference in treatment time scales (10 min vs. few milliseconds). In addition, in the centrifugal acceleration experiments, the cells partially recovered after 48 h, while after the dynamic impact, their viability decreased further over time. The energy-absorbing material could partially rescue the cellular viability, but not for the most severe impacts. Experimental and theoretical studies have shown that soft materials can reduce the impact severity only until they are fully compressed, and upon reaching that point, they can transmit shockwaves nearly as easily as hard materials can [44].

Taken together, these observations indicate that most of the irreversible phenomena in cells are the result of shockwaves emanating from the dynamic impact, while the g-force acceleration plays a less prominent role in irreversible cellular damage. As the Trypan Blue dye exclusion assay results revealed that the cell membrane remained largely intact even upon the hardest impact, the cell viability loss must have been due to internal cell damage.

Through PISA profiling, we found that the damage induced by dynamic impacts was likely related to the changes in the solubility of many cellular proteins, including those involved in the pathology of neurodegenerative diseases. This supported our previous findings on the alteration of the laminin structure by dynamic impacts [32]. As most of the detected solubility changes were positive (green and orange dots in Figure 4a), the disruption of protein complexes or organelles seemed to be the most likely damaging effect, while protein unfolding (red dots in Figure 4a) exacerbated the damage. This conclusion was supported by the pathway analysis of the most affected proteins that changed their solubility in proportion to the impact hardness.

The overall effect on proteome solubility was well represented by PC1 in PCA. These results open the way for the development of a proteome-based shock meter, which is conceptually shown in Figure 2c. Such a shock meter can be used to monitor the relative effects of dynamic impacts on neural cells under various circumstances and to help design more effective preventive measures, such as helmets and flooring materials. Cerebral concussions after kinetic energy impacts are the most common complications and are often defined as mild TBIs. At the same time, this is the most puzzling condition, as in many cases, they are characterized by different clinical symptoms [59]. Concussions currently have no generally accepted theoretical definition, as they cannot be monitored with the available imaging technologies. Thus, different kinds of TBIs can be theoretically graded with a proteome-based shock meter.

Low mechanical dynamic- and static-pressure forces can influence the structures of both proteins and protein complexes [60]. Tertiary and quaternary protein structures rely on hydrophobic interactions and hydrogen bonds to maintain their three-dimensional configuration for proper protein functioning. When these bonds are disrupted, e.g., under pressure or mechanical shock, the proteins can undergo unfolding events, which may be followed by misfolding [32,61]. Unfolded proteins usually lose their solubility, which can be measured with a PISA assay. While a distinct group of proteins seemed to unfold upon the dynamic impact, most proteins increased their solubility (Figure 3a and Figure 4a), which likely resulted from the breakage of the complexes in which they were involved. A scenario is also possible in which a protein in a complex unfolds upon an impact, which disrupts the complex and releases several proteins from it, thus increasing their solubility.

Soluble proteins are enshrined in a hydration shell that is often more dense than bulk water [62]. It is, thus, feasible that, as many proteins become more soluble after a dynamic impact, the overall volume of the hydration shells expands, necessitating water uptake from the extracellular and interstitial area, which results in the swelling of the cells in the TBI and, eventually, cytotoxic brain tissue edema.

Upon the breakage of the protein complex, the proteins may refold and form the complex again—in this case, the effect is reversible. We observed such an effect in the centrifugal acceleration experiments. The refolded proteins did not show marked solubility changes in the PISA analysis. On the other hand, the irreversible damage to proteins observed with the PISA assay after the hard impact is likely to have been responsible for triggering the cell death pathways responsible for continuous reduction in cellular viability between 24 h and 48 h after the impact.

The PISA- and proteome-based shock meter is likely to be reflective of irreversible cellular damage. Thus, using such a shock meter can help test and design energy-absorbing materials that reduce or prevent irreversible cell damage. Furthermore, the data presented in the current study will serve as a resource for follow-up studies on the acute and prolonged molecular consequences of massive proteome unfolding in TBIs, as well as its possible link to increased incidence of dementia and, potentially, other neurodegenerative disorders. Finally, the described proteome approaches may help in the development of innovative products for the primary prevention of TBI by providing protein targets for intervention and a means for assessing the preventive effect at the molecular level.

## 4. Materials and Methods

### 4.1. Cell Culture

Human SH-SY5Y cells (ATCC, Manassas, VA, USA) were grown at 37 °C in 5% CO_2_ using DMEM/F12 medium with GlutaMAX (cat#31331093, Thermo, Waltham, MA, USA) supplemented with 10% heat-inactivated FBS (cat#A3840402, Thermo, Waltham, MA, USA), 100 units/mL of penicillin/streptomycin (cat#15070063, Thermo, Waltham, MA, USA), MEM non-essential amino acids (cat#11140035), and HEPES buffer (cat#15630056, Thermo, Waltham, MA, USA). Low-number passages were used for the experiments.

### 4.2. Simulation of Dynamic Impacts at Different Heights

Cells were seeded at a density of 200,000 per well in Nunclon cell culture dishes (diameter 35 mm × 10 mm) and grown for 24 h. The dishes were then loaded into a dummy head with a weight of 5.1 kg, and an impact experiment was performed at different heights in 5 biological replicates. Cells that were not exposed to an impact were used as controls (controls were kept on the bench for the same duration of the dynamic impact experiment to minimize the effects of other variables). Depending on the experiment, cells underwent processing immediately or were kept in culture for different time periods before processing (the media volume was restored after the impact, but the detached cells were kept in culture).

### 4.3. Cell Viability Measurements

Cells were seeded at a density of 200,000 per well in Nunclon cell culture dishes and grown for 24 h. Impact experiments were performed similarly to those described above by dropping the dummy head from different heights. The control and treated samples were kept for 24 and 48 h. The cells that detached upon impact were kept in the dishes. Thereafter, cell viability was measured using a CellTiter-Blue Assay (Promega, Madison, WI, USA) according to the manufacturer’s protocol, as described previously [63].

To measure the viability of centrifuged cells, 250,000 cells were aliquoted in 300 μL PBS and centrifuged at 312, 625, 1250, 2500, 5000, 10,000, and 20,000× *g* for 10 min. Thereafter, the PBS was removed, fresh media were added, and cells were cultured in 6-well plates for 24 and 48 h. The viability measurement was performed as above.

### 4.4. PISA Assay

Cells were processed according to the PISA assay protocol [33]. After the impact, the media were removed, and cells were washed with PBS. An amount of 350 μL of PBS was added to each plate, after which the cells were scraped from the surface. Each sample was then divided into 10 aliquots on PCR plates. In the case of the centrifugation experiment, samples in PBS were directly aliquoted onto the PCR plates after centrifugation. The plate was heated in an Eppendorf gradient thermocycler (Mastercycler X50s, Eppendorf, Hamburg, Germany) in the temperature range of 48–59 °C for 3 min. Samples were cooled for 3 min at room temperature and then snap-frozen in liquid nitrogen. Samples from each replicate were then combined. The samples were freeze–thawed in liquid nitrogen twice more and then transferred into polycarbonate thick-wall tubes and centrifuged at 100,000× *g* and 4 °C for 20 min.

The soluble protein fraction was transferred to new Eppendorf tubes. Protein concentration was measured in all samples using a Pierce BCA Protein Assay Kit (Thermo, Waltham, MA, USA). The volume corresponding to 25 µg of protein was transferred from each sample to new tubes, and urea was added to a final concentration of 4 M. Dithiothreitol (DTT) was added to a final concentration of 10 mM, and samples were incubated for 1 h at room temperature. Subsequently, iodoacetamide (IAA) was added to a final concentration of 50 mM, and samples were incubated at room temperature for 1 h in the dark. The reaction was quenched by adding an additional 10 mM of DTT. The proteins were then precipitated using methanol/chloroform, and after drying, they were resuspended in 20 mM EPPS pH 8.5 with 8 M urea. Then, the urea was diluted to 4 M with 20 mM EPPS, and Lysyl endopeptidase (LysC; Wako, Fujifilm, Tokyo, Japan) was added at a 1:50 *w*/*w* ratio at room temperature overnight. Samples were diluted with 20 mM EPPS to the final urea concentration of 1 M, and trypsin was added at a 1:50 *w*/*w* ratio, followed by incubation for 6 h at room temperature. Acetonitrile (ACN) was added to a final concentration of 20%, and TMTpro-16plex reagents were added 4× by weight (200 μg) to each sample, followed by incubation for 2 h at room temperature. The reaction was quenched by the addition of 0.5% hydroxylamine. Samples within each replicate were combined, acidified with trifluoroacetic acid (TFA), cleaned using Sep-Pak cartridges (Waters), and dried using a DNA 120 concentrator (Thermo, Waltham, MA, USA). The pooled samples were resuspended in 20 mM ammonium hydroxide and separated into 96 fractions on an XBridge BEH C18 2.1 × 150 mm column (Waters; Cat#186003023) by using a Dionex Ultimate 3000 2DLC system (Thermo, Waltham, MA, USA) over a 48 min gradient of 1–63% B (B = 20 mM ammonium hydroxide in acetonitrile) in three steps (1–23.5% B in 42 min, 23.5–54% B in 4 min and then 54–63% B in 2 min) at a flow of 200 µL min^−1^. Fractions were then concatenated into 16 samples in sequential order. The PISA samples subjected to acceleration in the centrifuge were concatenated into 12 samples in sequential order after fractionation.

### 4.5. LC-MS/MS

After drying, the samples were dissolved in buffer A (0.1% formic acid and 2% ACN in water). The samples were loaded onto a 50 cm EASY-Spray column (75 µm internal diameter, packed with PepMap C18, 2 µm beads, 100 Å pore size) connected to a nanoflow Dionex UltiMate 3000 UHPLC system (Thermo, Waltham, MA, USA) and eluted in an organic solvent gradient that increased from 4% to 26% (B: 98% ACN, 0.1% FA, 2% H_2_O) at a flow rate of 300 nL min^−1^ over 180 min. The elution gradient steps were 4–5% B in 5 min, 5–28% B in 145 min, 28–34% B in 10 min, 34–95% B in 3 min, isocratic at 95% B for 5 min, 95–4% B in 2 min, and isocratic at 4% B for 10 min. The eluent was ionized with electrospray, and the mass spectra of the molecular ions were acquired with an Orbitrap Q Exactive HF mass spectrometer (Thermo, Waltham, MA, USA) in data-dependent mode at an MS1 resolution of 120,000 and an MS2 resolution of 60,000 in the *m*/*z* range from 375 to 1500. Peptide fragmentation was performed via higher-energy collision dissociation (HCD) with energy set at 33% NCE and an MS2 isolation width at 1.6 Th. The automatic gain control (AGC) targets were set at 3 × 10^6^ and 2 × 10^5^ for MS1 and MS2, respectively. The maximum injection time for ions (IT) was set to 100 ms in MS1 and 120 ms in MS2. The PISA samples subjected to acceleration in the centrifuge were analyzed using similar settings over a gradient lasting a total of 150 min. The elution gradient steps were isocratic 3% B for 6 min, 3–26% B in 115 min, 26–95% B in 10 min, isocratic at 95% B for 8 min, 95–3% B in 2 min, and isocratic at 3% B for 9 min.

### 4.6. Data Processing

The raw LC-MS data were analyzed with MaxQuant version 1.6.2.3 [64]. The Andromeda search engine [65] matched MS/MS data against the UniProt complete proteome database (human, version UP000005640_9606, 92,957 entries) unless otherwise specified. Trypsin/P was selected for enzyme specificity. No more than two missed cleavages were allowed. A 1% false discovery rate was used as a filter at both the protein and peptide levels. In all analyses, TMTpro 16-plex was used for peptide quantification. Cysteine carbamidomethylation was set as a fixed modification, while methionine oxidation and acetylation on N-termini were selected as a variable modification. For all other parameters, the default settings were used. After removing all the contaminants and proteins that were only identified by site, only proteins with at least two peptides were included in the final dataset. Proteins with missing values were removed, and protein intensities were normalized by total abundance in each TMT channel and then Log2 transformed. Protein fold changes were calculated by subtracting the Log2-transformed intensities.

### 4.7. Network Mapping

For pathway analyses, the STRING version 11.5 protein network analysis tool was used with the default parameters [66].

### 4.8. Statistical Analysis

Data analysis was performed using R project version 3.6.1. Analysis of significance was performed with a two-sided Student *t*-test between normalized intensities of impact vs. control replicates, and it is reported as such throughout the manuscript. PCA analysis was performed on normalized protein abundances. PC1 values for each sample (x coordinate) were extracted; the average position of the control cells on PC1 was taken as zero (reference point), and the coordinates of the cells after soft- and hard-floor impacts were calculated with respect to the reference point.

## Data Availability

The authors declare that all data supporting the findings of this study are available within the paper and its supplementary information files. All relevant data are available from the corresponding authors (H.V.H. and R.A.Z.). The mass spectrometry data that support the findings of this study have been deposited in ProteomeXchange Consortium (https://www.ebi.ac.uk/pride/, accessed on 19 September 2023) via the PRIDE partner repository [67] with the dataset identifiers PXD037675 (impact experiment) and PXD038607 (g experiment).

## Supplementary Materials

The following supporting information can be downloaded at: https://www.mdpi.com/article/10.3390/molecules28196768/s1, Table S1: Proteomics data of the cells exposed to dynamic impacts from 55 cm on the hard and soft floor.
